# Clinical decision-making in rare bone diseases – A survey among members of the European Paediatric Orthopaedic Society (EPOS) and the European Reference Network on Rare Bone Diseases (ERN BOND)

**DOI:** 10.1177/18632521261439074

**Published:** 2026-04-21

**Authors:** Thilini H. Gamage, Silvia Coutinho, Alberto Leardini, Inês Alves, Nadine Großmann, Ralph Sakkers, Gemma Marcucci, Laura Masi, Maria Luisa Brandi, M. Carola Zillikens, Giovanni Trisolino, Lena Lande Wekre, Joachim Horn

**Affiliations:** 1 Division of Orthopaedics, Section of Children’s Orthopaedics and Reconstructive Surgery, Oslo University Hospital, Oslo, Norway; 2 Norwegian Centre for Rare Diseases, Unit Sunnaas, Bjørnemyr, Norway; 3 Movement Analysis Laboratory, IRCCS Istituto Ortopedico Rizzoli, Bologna, Italy; 4 ANDO Portugal/ European reference Network for Rare Bone Diseases-ERN BOND, Evora, Portugal; 5 FOP e.V.,/International FOP Association (IFOPA)/ European reference Network for Rare Bone Diseases-ERN BOND, Berlin, Germany; 6 Free University of Berlin Institute of Chemistry and Biochemistry Berlin, Germany; 7 Berlin-Brandenburg School for Regenerative Therapies (BSRT), Charité – Universitätsmedizin Berlin, Berlin, Germany; 8 Orthopaedic Surgery, University Medical Centre Utrecht, Utrecht, the Netherlands; 9 Department of Experimental and Clinical Biomedical Sciences, University of Florence, Florence, Italy; 10 Metabolic Bone Diseases Unit, Musculoskeletal and Rehabilitation Department, University Hospital Careggi, Florence, Italy; 11 Fondazione FIRMO Onlus (Italian Foundation for the Research on Bone Diseases), Florence, Italy; 12 Endocrinology Unit, IRCCS San Raffaele Hospital, Milan, Italy; 13 Department of Internal Medicine, Erasmus MC, Erasmus MC University Medical Center Rotterdam, Rotterdam, the Netherlands; 14 Pediatric Orthopedics and Traumatology, IRCCS Istituto Ortopedico Rizzoli, Bologna, Italy; 15 Institute for Clinical Medicine, University of Oslo, Oslo, Norway

**Keywords:** Clinical practice, rare bone diseases, rare diseases, clinical practice survey, bone disorder

## Abstract

**Purpose:**

Clinical management of rare bone diseases (RBDs) is challenged by low prevalence, delayed diagnosis, and complex multidisciplinary needs. This survey aimed to map current clinical practices in RBDs, identify unmet needs, and generate foundational data to guide the development of minimum standards for patient assessment.

**Methods:**

An anonymous online survey was distributed to members of the European Paediatric Orthopaedic Society (EPOS) and the European Reference Network on Rare Bone Diseases (ERN BOND) in September 2025. Questions addressed diagnostic work-up, interdisciplinary care, transition practices, and future perspectives. Quantitative data were analysed descriptively, and free-text responses thematically.

**Results:**

A total of 119 respondents (35 countries), mostly orthopaedic surgeons (74%), completed the survey. Almost all (118/119) provided direct care, and 63% had >10 years’ experience treating RBDs. Over 80% routinely used anthropometric, posture, and alignment measures, whereas the use of advanced tools varied. Interdisciplinary care is widely applied at varying frequencies, with a high consideration for shared decision-making and quality of life. Most lacked registry access (>80%) and formal transition protocols (~70%). Respondents prioritised clinical frameworks over technological advances and anticipated increasing future relevance for technological innovations.

**Conclusions:**

This survey highlights considerable variability in clinical decision-making for RBDs. The findings underscore the importance and need of standardised interdisciplinary care, registry data, and structured protocols and frameworks.

**Study Significance:**

This is the first systematic survey of clinical practice and decision-making process in RBD care among EPOS and ERN BOND members. The findings may guide future recommendations and standards, supporting more harmonised care for individuals with RBDs, especially ultra-rare conditions.

## Introduction

Rare bone diseases (RBDs) encompass more than 700 distinct conditions, each characterised by low prevalence, phenotypic variability, and complex clinical trajectories.^1–5^ Their rarity often results in diagnostic delays, fragmented care, and limited clinician experience, which in turn contribute to inconsistent decision-making and variable patient outcomes.^6,7^ Effective management of these disorders frequently requires interdisciplinary collaboration and patient-centred approaches, but evidence-based guidance remains sparse.^8–10^

The European Reference Network on Rare Bone Diseases (ERN BOND) was established to address these challenges by gathering expert centres across Europe and working toward a ‘common minimum standard’ for patient assessment and care.^11,12^ Similarly, the European Paediatric Orthopaedic Society (EPOS) has been increasingly engaged in advancing knowledge and developing frameworks for the management of skeletal dysplasias and other rare bone conditions.^13^ Despite these initiatives, little is still known about how healthcare professionals currently make clinical decisions for RBD management in real-world healthcare practice.

To address this gap, we conducted an online survey among members of EPOS and ERN BOND. The survey was designed to capture current clinical practices, decision-making strategies, use of diagnostic tools such as gait analysis, interdisciplinary collaborations, and approaches in transition of care. Furthermore, the survey aimed to identify unmet needs and educational priorities as a foundation for future guideline development. By mapping current practices, this study offers preliminary insights that can support the harmonisation of care and the development of decision-making frameworks for RBD management.

## Methods

### Study design

An anonymous, cross-sectional online survey was conducted using the Google Forms platform (Google LLC, USA) (Supplemental File 1: Survey). The questionnaire combined multiple-choice, Likert-scale, and open-ended questions, facilitating both quantitative and qualitative data collection. Optional free-text responses were included to allow participants to elaborate on their practices, challenges, and recommendations beyond predefined options. Participation was voluntary, and no personal identifiers were collected, ensuring respondent anonymity.

### Study population

The target population comprised healthcare professionals and researchers with experience in RBDs; specifically, EPOS and ERN BOND members, engaged in the diagnosis, treatment, and follow-up of patients with RBDs.

On dissemination, the survey invitation further encouraged individuals with experience in RBD care to participate. A self-evaluated (yes/no) screening question at the start of the survey ensured that only respondents involved in RBD care, irrespective of the extent of experience, were able to proceed and complete the full questionnaire (Supplemental File 1: Survey).

### Survey instrument

The questionnaire was available in English and was structured to be completed within 10–15 min (Supplemental File 1: Survey). The survey was developed collaboratively by clinical RBD experts in orthopaedics, endocrinology, and gait analysis, in partnership with patient representatives from ERN BOND. The survey was elaborated as part of the aims of working package 7 (WP7-Clinical Practice Guidelines and Clinical Decisions support tools) of ERN BOND, aligning with the ERN’s mission to define a common minimum standard for the assessment of patients with RBDs across Europe. Dissemination was set under the collaborative framework between EPOS and ERN BOND. It underwent two rounds of pilot testing and peer review to ensure clarity, inclusivity, and reduction of bias in question wording and answer options.

The survey was divided into seven domains (Supplemental File 1: Survey):

1. Background information – This section surveys respondents’ demographics, professional background, level of RBD experience, country and primary practice setting, and the types of RBDs they have worked with.
2. Diagnostic workup and decision-making – Given the heterogeneity of RBDs, diagnosis requires integrated clinical, imaging, genetic evaluation and a systematic interdisciplinary approach.^1^ This section surveyed the frequency of diagnostic assessments used (‘routinely’, ‘as needed’ or ‘never’), interdisciplinary collaboration and related challenges.
3. Research, natural history and long-term data – Registry data are essential in understanding the disease course, progression and risk factors, which are often poorly understood for rare conditions.^12^ This section surveyed the availability and the use of registries and natural history data, and their role in the current practice and clinical decision-making in RBDs.
4. Role of gait analysis – Gait analysis tools provide an objective evaluation and are perceived valuable in several aspects of RBD care.^3^ This section surveyed the types of gait analysis tools being used, their perceived effectiveness and limitations in assessing RBDs.
5. Future perspectives – This section surveyed unmet needs, resource requirements, and the perceived role of emerging tools, including digital and AI-based solutions, to inform priorities for protocols, standards, and guidelines.
6. Shared/patient centred decision-making – This section examined how socioeconomic and cultural factors, along with patient representatives and organisations, influence collaboration and shared decision-making between patients and their healthcare providers in RBD care.
7. Transition of care – Transition in care require established strategies and protocols,^14^ and this section surveyed current strategies and the need for further recommendations and/or guidelines to strengthen transition from paediatric to adult care in RBDs.

### Survey distribution

The survey was distributed electronically to all EPOS and ERN BOND members via official mailing lists. A minimum of 850 members received the email invitation for participation followed by 2 reminders at the end of the first and second weeks. The survey remained open for 3 weeks in September 2025.

### Data analysis

Quantitative data were analysed using descriptive statistics, with frequencies and proportions calculated for categorical and ordinal responses and presented as percentages supplemented by tables and graphs. Likert-scale items were illustrated using stacked bar charts and summary statistics. Qualitative and free-text responses were analysed thematically by two independent reviewers, identifying recurring patterns and grouping into themes, and discrepancies were resolved through discussion with a third reviewer.

### Ethical considerations

The survey posed minimal risk to participants, as no personal or identifiable information was collected. Survey participation was voluntary, and respondents could exit the survey any time. Responses were saved only upon final submission, which implicated informed consent. Responses were stored securely on Google’s servers and accessed only by the study team. Ethical approval was not required under European regulations for anonymous survey-based studies of healthcare professionals.

## Results

### Background information

**Figure 1.**
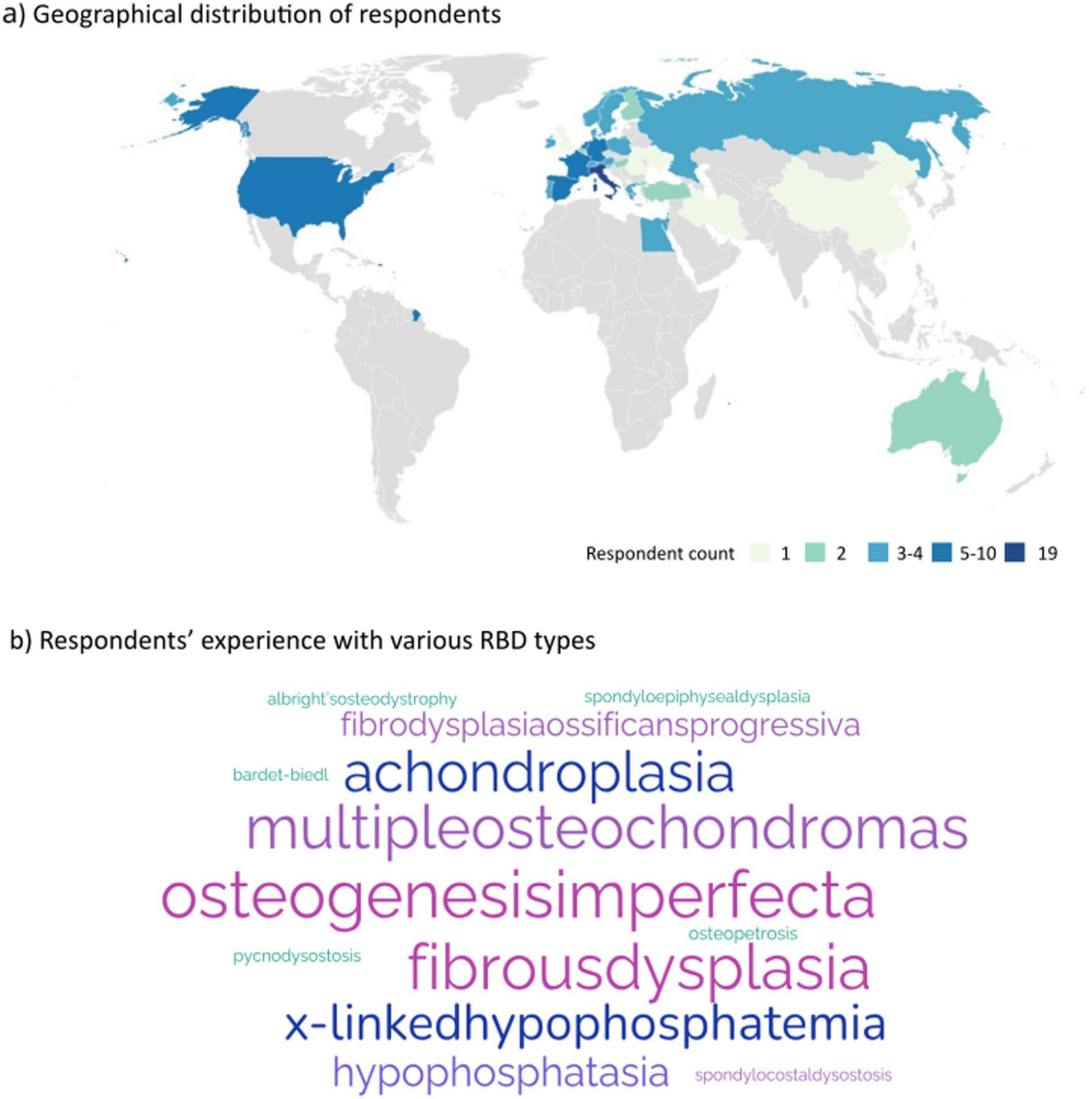
Respondent demographics. (a) Map indicating the representative countries of respondents. The colour legend indicates the number of respondents per country. (b) A word cloud illustrating respondents’ experience with various RBD types; larger font size indicates greater number of respondents with experience treating the condition. RBD: Rare bone diseases.

A total of 134 respondents initiated the survey. Following a self-screening question for involvement in RBD care; 119 eligible respondents from 35 countries continued to completion. The highest participation was from Italy (19/119, 16%), followed by the Netherlands, Spain, Germany, France, Israel, and the United States, each contributing with 5–10 responses (Figure 1(a)).

Orthopaedic surgery was the most represented specialty (88/119, 73.9%), followed by endocrinology (17/119, 14.3%) and paediatrics (12/119, 10,1%). Several orthopaedic surgeons reported dual roles/specialities (7/88, 8%), including paediatric orthopaedics (1/88, 1.1%), gait analysis (2/88, 2.3%), rehabilitation (1/88, 1.1%), or research (3/88, 3.4%). Most paediatricians (10/12, 83.3%) held multi-speciality roles, for example, in paediatric endocrinology (7/12, 58,3%), genetics (2/12, 16.7%) or research (3/12, 25%). Overall, only 3 respondents were rheumatologists (3/119, 2.5%), while 1 radiologist (1/119, 0.8%), 1 internist (1/119, 0.8%), 1 geriatrician (1/119, 0.8%), and 4 geneticists (4/119, 3.4%) also participated. Researchers were represented in both clinical and non-clinical capacities.

Almost all (118/119, 99.2%) were directly involved in clinical care of RBD patients, with 62% reporting over 10 years of experience. Beyond clinical care, 68 respondents (68/119, 57.1%) contributed to RBD management in multiple capacities; with nearly half (56/119, 47.1%) engaged in research, while others were involved in laboratory based roles (31/119, 26%) (genetic diagnostics, imaging/biomechanical assessments), advocacy, rehabilitation, policymaking, and leadership roles (24/119, 20.2%) with several (7/119, 5.9%) contributing in 5–7 of these distinct aspects in RBD care.

Most respondents (81/119, 68.1%) were based in university hospitals or academic centres, while 12% (14/119) worked in general hospitals and 10% (12/119) in specialty hospitals. The remaining were affiliated with private institutions, research centres, or rehabilitation centres (12/119, 10.1%).

Common RBDs-osteogenesis imperfecta, fibrous dysplasia, achondroplasia, and multiple osteochondromas accounted for 70.7% of all optional diagnoses the respondents had most experience with, while experience with ultra-rare RBDs was less frequent (29.3%) (Figure 1(b), Table 1).

**Table 1.** Respondents’ experience with various RBDs; the frequency indicates the number of respondents having experience with the specified RBD.

Category	RBD	Frequency
Rare	Osteogenesis imperfecta	109
	Fibrous dysplasia	104
	Multiple osteochondromas	98
	Achondroplasia	89
	X-linked hypophosphatemia	76
	Osteopetrosis	3
	Bardet-Biedl	1
	Albright’s osteodystrophy	1
	Spondyloepiphyseal dysplasia	1
Ultra-rare	Hypophosphatasia	54
	Fibrodysplasia ossificans progressiva	36
	Pycnodysostosis	1
	Spondylocostal dysostosis	1

RBD: Rare bone disease.

### Diagnostic work-up and decision-making

**Figure 2.**
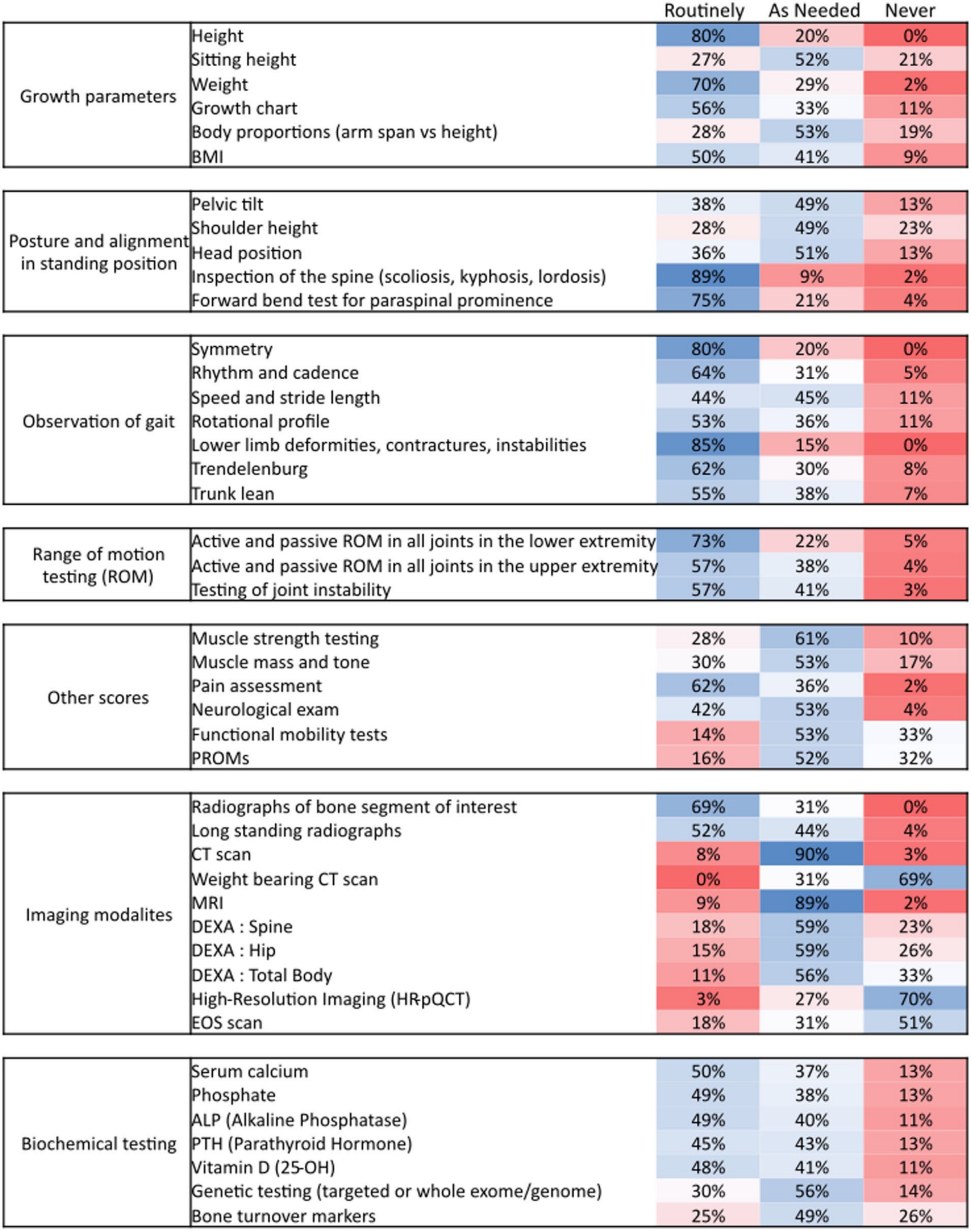
Use and the frequency of use of clinical parameters for disease assessment in RBDs. BMI: Body mass index; PROMs: Patient reported outcome measures; CT: Computed tomography scan; MRI: Magnetic resonance imaging; DEXA: dual-energy X-ray absorptiometry.

Among the parameters, height, weight, growth chart, body mass index, spinal inspections, gait observation parameters, range of motion (ROM) testing (lower extremity, upper extremity, spinal/axial), pain assessment, were found routinely used by at least 50% of respondents during the diagnostic workup of RBDs. Sitting height, body proportions, standing posture alignment, muscle mass, tone and strength, neurological exams are also used ‘as needed’ by at least half of the respondents (>49%) (Figure 2). Additional assessments included head circumference/centile, limb length, lower limb alignment, and bone age.

Functional mobility tests were found to be mostly used as required. About half of the respondents (57/119, 47.9%) specified the tests used. The most common were the 6-Minute Walk Test (6-MWT, 18/57, 31.6%) and Timed Up and Go (TUG, 15/57, 26.3%). Other tests mentioned included the 10-Meter Walk Test (10-MWT), Timed Up and Down Stairs, Single Leg Squat, Toe-Heel Walking, Functional Mobility Scale, Pirani Böhm Sinclair clubfoot score, Gross Motor Function Measure (GMFM-66/GMFM-88), and handheld dynamometry.

Only 16% of respondents (18/119) reported to routinely use patient-reported outcome measures (PROMs), while 52% (59/119) used them as needed. Commonly used PROMs included the Visual Analogue Scale (18/67, 26.9%), EuroQol-5D (EQ-5D, 7/67, 10.4%), Paediatric Quality of Life Inventory (6/67, 9%), and 36-Item Short Form Health Survey (5/67, 7.5%). Several respondents (9/119, 7.6%) also relied on clinical observations and direct patient communication as alternatives.

**Figure 3.**
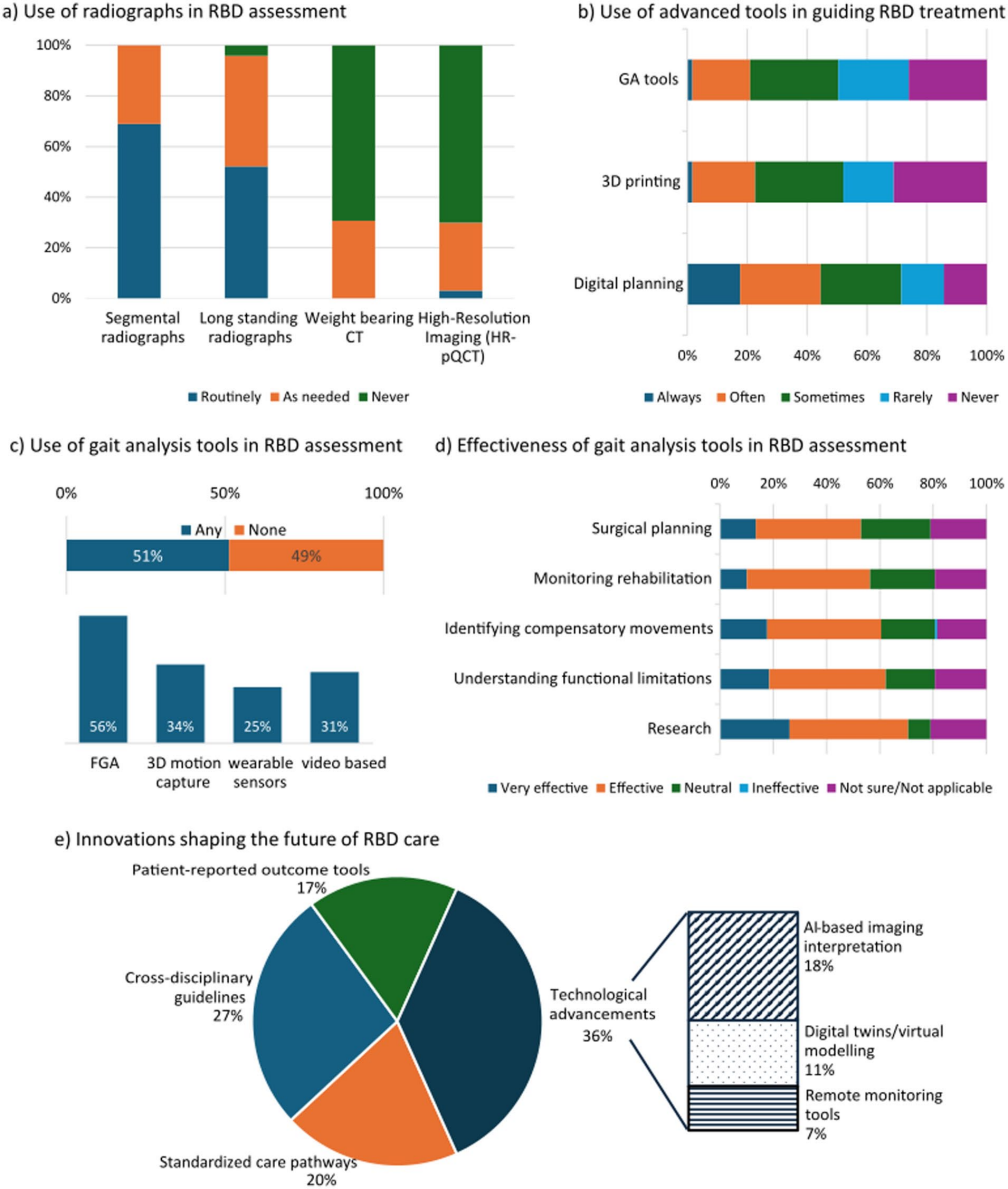
Use of radiology, gait analysis and other advanced tools and future perspectives in RBD care. High variability in the frequency of use of (a) segmental and long-standing radiographs versus weight-bearing CT and high-resolution imaging and (b) GA tools, 3D printing and digital planning in routine RBD care (c) GA tools and their popularity for RBD assessment (d) The perceived effectiveness of GA tools different aspects of RBD care (e) Innovations considered relevant for the future of RBD care; the bar-of-pie chart shows overall categories of anticipated innovations, while the bar chart (right) details a breakdown of the anticipated technological advancements. RBD: Rare bone disease; GA: Gait analysis; CT: Computed tomography.

Segmental and long-standing radiographs were the imaging modalities reported as routinely used by at least 52%, whereas Computed tomography (CT) and Magnetic resonance imaging (MRI) were mostly used as needed (>89%). Weight-bearing CT, high-resolution imaging, and EOS imaging were reported to be rarely or never used (Figure 2, Figure 3(a)).

Biochemical parameters (serum calcium, serum phosphate, alkaline phosphatase, parathyroid hormone, and 25-OH vitamin D) were measured either routinely or as needed by most respondents (>87%), while genetics and bone turnover markers were primarily tested as needed (≥49%) (Figure 2).

All respondents reported the availability of an interdisciplinary approach in their clinical practice in RBD management. However, the frequency of interdisciplinary case discussions varied: 19.3% (23/119) held weekly meetings, 36% (43/119) monthly, while 43% met only occasionally or when the need arose.

Of 11 listed specialties (orthopaedics, endocrinology, paediatrics, genetics, radiology, rehabilitation medicine, neurology, rheumatology, psychology, physiotherapy, and occupational therapy), the first five listed, were reported as the core specialities in the interdisciplinary RBD care, each including in >75% of responses. A combination of 5–8 specialities was commonly mentioned (83/119, 69.7% respondents). Rehabilitation and physiotherapy were also reported as common adjuncts, while neurological, psychological, and occupational specialties were less frequent (20%–40% involvement).

### Research, natural history and long-term data

Nearly all respondents considered long-term follow-up studies and natural history studies either ‘very’ or ‘extremely’ important for guiding treatment decisions, and over 90% often or always considered long-term outcomes. However, only 19% (23/119) had access to national or international registry data, 37% (44/119) to local/institutional databases, and 32.8% (39/119) relied on published long-term follow-up studies while 10.9% (13/119) reported no access to long-term data.

### Role of Gait analysis

The reported use of gait analysis tools was split, with 51% (61/119) reporting to use any type of gait analysis tools (Figure 3(c), top). Of the subgroup of orthopaedic surgeons, rheumatologists and radiologist professionals (92/119), only 55% (51/92) reported to use gait analysis tools.

Only 21% of respondents (23/119) and 25% of the above subgroup (23/92) reported that gait analysis ‘often’ or ‘always’ influenced treatment, ~30% noted occasional impact, while, for about half, it rarely or never influenced their clinical decisions (Figure 3(b)). Among the 61 users, most employed fully instrumented gait analysis (FGA, 34/61, 55.7%), with 3D motion capture, wearable sensors, and video/AI methods used by 25%–35% (Figure 3(c), bottom).

Despite limited influence on practice, more than half recognised gait analysis as valuable for surgical planning (63/119, 52.1%), monitoring rehabilitation progress (67/119, 56.3%), identifying compensatory gait patterns (72/119, 60.5%), and understanding functional limitations (74/119, 62.2%). The greatest perceived benefit was in research, with 71% (84/119) identifying it as highly effective for scientific advancements (Figure 3(d)).

The main challenges in implementing gait analysis were costs and limited access (82/119, 68.9%), high time and resource demands (79/119, 66.4%), lack of guidelines (48/119, 40.3%), and variable relevance across RBD types (42/119, 35.3%), with patient compliance being least reported (24/119, 20.2%).

Respondents recognised gait analysis as most beneficial for patients with lower limb deformities (84/119, 70.6%) and pre-/post-operative surgical evaluation (78/119, 65.5%). It was also considered useful for identifying static/dynamic discrepancies (57/119, 47.9%), and for moderate to severe mobility impairment with postural control issues (59/119, 49.6%), but less useful for severe functional impairments (39/119, 32.8%) or rapidly progressing RBDs (19/119, 16%).

### Future perspectives

Digital planning was reported as being used more frequently (53/119, 44.4%) than 3D printing (27/119, 22.6%) in clinical settings. Almost half (57/119, 47.9%) reported rare (16.8%) or absent (31.1%) use of 3D printing for deformity assessment or treatment planning.

Respondents anticipated growing importance of both digital and technological innovations (i.e. AI-based imaging interpretation, digital twins/virtual modelling, and remote monitoring tools) and clinical and patient-centred care frameworks (i.e. standardised care pathways, cross-disciplinary guidelines, PROMs), with the latter prioritised with 63.4%. Cross-disciplinary guidelines (80/119, 26.8%) and standardised care pathways (59/119, 19.8%) were the most anticipated, followed by AI-based imaging (54/119, 18.1%) and PROMs (50/119, 16.8%), while digital twins (33/119, 11.1%) and remote monitoring tools (22/119, 7.4%) were less prioritised (Figure 3(e)).

### Shared/patient-centred decision-making

**Figure 4.**
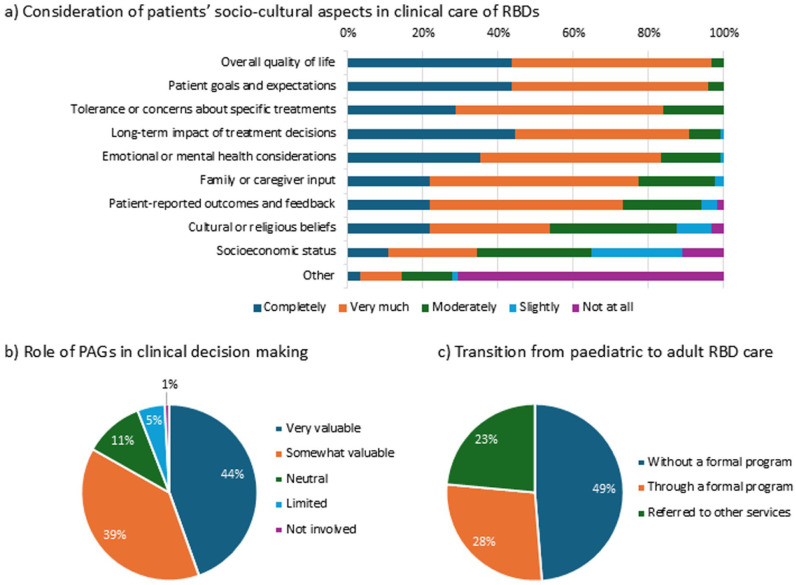
Shared decision-making, role of patient representative organisations, care transition. (a) Consideration and influence of various socio-cultural aspects of RBD patients in clinical decision-making (b) Perceived importance of the role of PAGs in RBD care (c) Current practice of transition of care from paediatric to adult setting in RBDs. RBD: Rare bone disease; PAG: Patient advocacy group.

The respondents were also requested to rate the level of consideration for a list of socio-cultural aspects. Almost all were highly considerate of overall quality of life and patient goals and expectations (>95%), as well as the long-term impact of treatment decisions (108/119, 90.7%). Tolerance and other treatment concerns as well as emotional or mental health were also among the highly considered aspects (82%–83%). Family and caregiver input, PROMs and feedback were also considered (~75%). The least considered measures were cultural and religious beliefs (64/119, 53.8%) and socioeconomic statuses (41/119, 34.4%) of the patients (Figure 4(a)).

The value of the role of patient representative organisations and advocacy groups in clinical decision-making in RBDs, was acknowledged by 83.2% (44.5% ‘very valuable’, and 38.7% ‘somewhat valuable’) (Figure 4(b)).

### Transition of care from paediatric to adult services

The majority (102/119, 85.7%) considered a formal structured tool for continuity of care (transition care) in RBDs important, of which 60% (71/119) rated it as extremely important. However, only 27.7% (33/119) reported having an established formal transition care programme. While nearly half (58/119, 48.7%) provided continuous care without a formal programme, the remaining 23.5% (28/119) referred patients to other services (Figure 4(c)).

Several challenges persist when transitioning patients with RBDs from paediatric to adult care, including poor communication/collaboration between paediatric and adult teams, limited transition guidelines or protocols, loss to follow-up or patient disengagement, geographical barriers (e.g. different geographic locations, long travel distances to adult centres), and limited availability of multidisciplinary teams in adult services; of which the most common were patient disengagement (74/119, 62.2%) and limited availability of multidisciplinary teams in adult care services (73/119, 61.3%). Over a quarter of respondents (31/119, 26.1%) named 4 or more challenges while 56.3% (67/119) named at least 3 challenges being faced during the care transition in RBDs. The most common combination was poor communication/collaboration between paediatric and adult teams, patient disengagement, and limited availability of multidisciplinary resources. In addition, limitations including lack of financial/charitable resources for transition care in RBDs and lack of parental support in adulthood were also highlighted.

## Discussion

This survey provides the first systematic mapping of clinical decision-making practices in RBDs among members of EPOS and ERN BOND. Responses revealed substantial heterogeneity in clinical assessment strategies, tool utilisation and care pathways across European and some non-European nations. Although most respondents work in tertiary care units with access to multidisciplinary teams, considerable variability persists in the availability and use of key resources. This includes structured transition pathways, registry data, interdisciplinary care, and advanced diagnostic tools such as FGA. This aligns with previous literature, highlighting the lack of standardised protocols in RBD care^7,15^ and underscores the persistent gap between recognised value and real-world implementation.

Although the survey was sent to all 850 EPOS and ERN BOND members, the exact number of members with specific RBD experience is unknown. Participation was encouraged only for those with RBD experience and was further limited to eligible respondents via a self-screening question, making the precise response rate indeterminable. As surveys of specialised clinical communities typically yield modest responses,^16^ lower numbers can be expected for a further sub-specialised and niche field like RBD. In this context, the absolute response number, for example, 119 responses from 35 countries, can represent a substantial and meaningful dataset, capturing a considerable proportion of experts actively managing RBDs with broad geographical representation.

The variability between the use of core clinical measurements (e.g. growth parameters, posture/alignment, ROM) and routine radiography in comparison to CT, MRI, and other advanced imaging techniques (e.g. EOS, weight-bearing CT) reflects on the individualised, condition-specific diagnostic approach typical for RBDs, while the latter may also reveal possible infrastructure limitations.

Given the rarity and lifelong nature of RBDs, standardised national and international registry data are essential for tracking disease progression and evaluating the effectiveness of treatment strategies. Despite the near-universal agreement on their importance, limited access in many centres, highlights a clear opportunity for advancement by improving availability and accessibility to datasets, and to provide pragmatic longitudinal frameworks. The European Registries for Rare Endocrine and Bone represents a major step toward addressing this deficit.^11^ However, registry participation is often limited by local data entry requirements and ethical approvals, likely explaining respondents’ limited access.

Respondents viewed FGA to be most beneficial for patients with lower limb deformities or undergoing surgery, while its perceived value decreased with greater functional impairment or rapidly progressing disease, highlighting its selective applicability based on disease stage and mobility. Although about half of respondents used FGA, few reported it regularly influenced treatment decisions. Nonetheless, it was still valued for surgical planning, rehabilitation monitoring, and research, with systemic barriers (e.g. availability, costs, personnel, and time) limiting its broader use, as previously described.^17^ Improved access supported through investments in specialised centres and regional organisation models such as hub-and-spoke models, may improve usage by enabling shared expertise and resources across centres.

All centres reported access to interdisciplinary collaboration, although only a subset met routinely. Establishing minimum standards for required specialties, meeting frequency, and documentation and communication systems within these teams for interdisciplinary RBD care could reduce variability and support more efficient programme development. The Clinical Patient Management System [https://ernbond.eu/cpms-2/] is one such initiative introduced by the European commission, as a secure web-based platform virtually connecting experts for multidisciplinary discussions on rare and complex cases supporting cross-border diagnosis, care, and treatment.^18,19^ This facilitates collaboration, data sharing, and expert consultation, and allows external clinicians to join panels or enrol patients for specialised advice.^18,19^

Another critical gap concerns the transition of care, as most respondents, despite working in university hospitals, reported the absence of formalised transition protocols. Given the strong consensus in the survey on the importance of transition tools in preventing loss to follow-up and clinical deterioration in adulthood, a simple transition checklist could provide rapid improvement awaiting development and evaluation of comprehensive pathways, an area in which harmonised strategies could have a substantial impact.^20^

Furthermore, two systematic reviews on RBD transition identified key success factors: co-ordinated multidisciplinary care with a lead clinician, individualised transition plans, attention to changing priorities in young adulthood, strong communication and shared decision-making, and supportive tools such as transfer summaries and digital self-management resources.^14,21^ Together, these elements may form the core of successful paediatric-to-adult care transition models for RBDs.

Patient-centred decision-making in RBD care involves collaboration between patients and healthcare providers to tailor treatment to each patient’s unique clinical and psychosocial needs. The strong positive responses in this survey indicate that these practices are well integrated in the care practices among respondents. Nevertheless, more time and access to multidisciplinary resources, evidence-based decision aids, language and culturally adapted material resources for patient education, were deemed necessary, also to further enhance the collaborative decision-making.

Collaboration with patient representatives, organisations and advocacy groups is deemed very valuable among the survey respondents (>80%). They play a key role in identifying research priorities, representing patient perspectives in guideline development and research design and raising awareness through educational initiatives.^22^ Strengthening social support systems not only enhances coping abilities but also provides support, education, and a community for affected individuals, in turn improving access to resources and promoting adherence to medical recommendations which can significantly improve disease management.^23^ The comparatively lower consideration of patients’ socioeconomic status in clinical decision-making among our respondents may reflect the respondents’ practice settings, with a majority being based in university hospitals and academic centres with public funding where financial burden on patients is minimised.

For future directions, respondents prioritised developing cross-disciplinary guidelines and standardised care pathways over emerging technologies like digital twins or 3D printing. Establishing internationally validated, cross-disciplinary guidelines and standardised care pathways is critical for managing RBDs. For example, in multiple osteochondromas, phenotype-based classifications show limited correlation with natural history, treatment indications, and or even basic follow-up requirements, underscoring the need for care pathways updated with evidence-based recommendations and expert consensus including imaging timing and modality.^24^ Similarly, in osteogenesis imperfecta and achondroplasia, the advent of new therapies has revealed substantial variation across major European centres in monitoring, medical protocols, pharmacological management, and surgical decision-making.^25–27^ These gaps highlight the urgent need for robust, consensus-driven standards for RBD management.

Although priority has been given to developing and adopting RBD-specific guidelines, recommendations, and treatment protocols, there is a growing overall interest in introducing novel technologies such as digital surgical planning and AI-assisted imaging. Recent studies have demonstrated the feasibility and effectiveness of virtual surgical planning and computer-assisted deformity correction, particularly in paediatric rare diseases.^28,29^ Combined with in-house 3D printing, these approaches improve precision, safety, and efficacy, even in complex deformities.

A pragmatic roadmap could therefore combine: (1) core clinical datasets to be recorded in all centres, (2) shared decision-making tools and effective communication strategies based on interdisciplinary care, tailored to RBDs, (3) structured transition guidelines, and (4) selective implementation of technologies that may enhance patient management, such as FGA, 3D labs, digital twins, and AI-driven tools for diagnosis, disease monitoring, or treatment simulation for specific indications.

## Strengths and limitations

This survey’s strengths include a broad international sample from EPOS and ERN BOND and detailed data across the spectrum of RBD care, supporting the validity and relevance of findings for guideline development and identifying improvement priorities.

Despite its strengths, several limitations must be acknowledged. These include voluntary participation, potentially biasing responses toward clinicians more engaged in RBD care; overrepresentation of some countries, particularly Italy, in this survey; small representation from certain specialties (radiology, rheumatology, genetics), limiting cross-disciplinary comparisons; and reliance on self-reported practices, subjecting to recall bias. As the survey addressed RBDs as a single category, the responses mainly reflect experience with the conditions most familiar to respondents and should be interpreted accounting for these factors.

## Conclusions

Overall, this survey provides an overview of current practice and unmet needs in RBD management. Experts recognise the importance of interdisciplinary care, longitudinal data collection, and structured transition planning, yet implementation remains uneven, particularly in the adoption of newer technologies, as well as in the establishment of formal protocols and standardised guidelines. Harmonised strategies-defining minimum standards for interdisciplinary care, shared decision-making tools, practical guidance for FGA use and reporting, and streamlined transition protocols could substantially improve consistency and quality of care. These findings highlight actionable priorities for ERN BOND and EPOS and will inform future work evaluating the uptake and clinical impact of new care pathways and technological innovations.

## Supplemental Material

sj-pdf-1-cho-10.1177_18632521261439074 – Supplemental material for Clinical decision-making in rare bone diseases – A survey among members of the European Paediatric Orthopaedic Society (EPOS) and the European Reference Network on Rare Bone Diseases (ERN BOND) Supplemental material, sj-pdf-1-cho-10.1177_18632521261439074 for Clinical decision-making in rare bone diseases – A survey among members of the European Paediatric Orthopaedic Society (EPOS) and the European Reference Network on Rare Bone Diseases (ERN BOND) by Thilini H. Gamage, Silvia Coutinho, Alberto Leardini, Inês Alves, Nadine Großmann, Ralph Sakkers, Gemma Marcucci, Laura Masi, Maria Luisa Brandi, M. Carola Zillikens, Giovanni Trisolino, Lena Lande Wekre and Joachim Horn in Journal of Children's Orthopaedics

sj-pdf-2-cho-10.1177_18632521261439074 – Supplemental material for Clinical decision-making in rare bone diseases – A survey among members of the European Paediatric Orthopaedic Society (EPOS) and the European Reference Network on Rare Bone Diseases (ERN BOND) Supplemental material, sj-pdf-2-cho-10.1177_18632521261439074 for Clinical decision-making in rare bone diseases – A survey among members of the European Paediatric Orthopaedic Society (EPOS) and the European Reference Network on Rare Bone Diseases (ERN BOND) by Thilini H. Gamage, Silvia Coutinho, Alberto Leardini, Inês Alves, Nadine Großmann, Ralph Sakkers, Gemma Marcucci, Laura Masi, Maria Luisa Brandi, M. Carola Zillikens, Giovanni Trisolino, Lena Lande Wekre and Joachim Horn in Journal of Children's Orthopaedics

## References

[1] Unger S Ferreira CR Mortier GR , et al. Nosology of genetic skeletal disorders: 2023 revision. Am J Med Genet A 2023; 191: 1164–1209. 36779427 10.1002/ajmg.a.63132 PMC10081954

[bibr2-18632521261439074] Alves I Westerheim I Hsiao EC , et al. A systematic literature review of the impact and measurement of mobility impairment in rare bone diseases. Ther Adv Musculoskelet Dis 2025; 17: 1759720x251369963. 10.1177/1759720X251369963 PMC12374048 40862204

[bibr3-18632521261439074] Horn J Leardini A Benedetti MG , et al. Fully instrumented gait analysis in rare bone diseases – a scoping review of the literature. Gait Posture 2025; 118: 168–177. 39978051 10.1016/j.gaitpost.2025.02.001

[bibr4-18632521261439074] Seefried L Smyth M Keen R , et al. Burden of disease associated with X-linked hypophosphataemia in adults: a systematic literature review. Osteoporos Int 2021; 32: 7–22. 32710160 10.1007/s00198-020-05548-0 PMC7755619

[5] Westerheim I Hart T van Welzenis T , et al. The IMPACT survey: a mixed methods study to understand the experience of children, adolescents and adults with osteogenesis imperfecta and their caregivers. Orphanet J Rare Dis 2024; 19: 128. 38515144 10.1186/s13023-024-03126-9 PMC10956293

[6] Phillips C Parkinson A Namsrai T , et al. Time to diagnosis for a rare disease: managing medical uncertainty. A qualitative study. Orphanet J Rare Dis 2024; 19: 297. 39143641 10.1186/s13023-024-03319-2 PMC11323401

[7] Chandran M Alves I Carpenter T , et al. Improving care pathways for people living with rare bone diseases (RBDs): outcomes from the first RBD Summit. Osteoporos Int 2023; 34: 1301–1310. 37294334 10.1007/s00198-023-06791-x PMC10382343

[8] Mindler GT Stauffer A Chiari C , et al. Achondroplasia current concept of orthopaedic management. J Child Orthop 2024; 18: 461–476. 39391573 10.1177/18632521241269340 PMC11463089

[bibr9-18632521261439074] Shore EM Pacifici M. JBMRPlus: special issue on rare bone diseases 2019. JBMR Plus 2019; 3: e10218. 10.1002/jbm4.10218 PMC6715777 31485556

[10] Aartsma-Rus A Dooms M Le Cam Y. Orphan medicine incentives: how to address the unmet needs of rare disease patients by optimizing the European orphan medicinal product landscape guiding principles and policy proposals by the European expert group for orphan drug incentives (OD Expert Group). Front Pharmacol 2021; 12: 744532. 34975469 10.3389/fphar.2021.744532 PMC8717920

[11] Casareto L Appelman-Dijkstra NM Brandi ML , et al. ERN BOND: the key European network leveraging diagnosis, research, and treatment for rare bone conditions. Eur J Med Genet 2024; 68: 104916. 38296035 10.1016/j.ejmg.2024.104916

[12] Priego Zurita AL Boarini M Casareto L , et al. The role of the European Reference Network for Rare Bone Diseases (ERN BOND) and European Registries for Rare Bone and Mineral Conditions (EuRR-Bone) in the governance of the management of rare bone and mineral diseases. Calcif Tissue Int 2024; 115: 498–506. 39060404 10.1007/s00223-024-01256-7 PMC11531435

[13] EPOS-Genetics and Metabolic study group, https://www.epos.org/governance/study-groups (accessed 8 December 2025).

[14] Grasemann C Wernsmann J Appelman-Dijkstra NM , et al. Transition care for young persons with rare bone mineral conditions: a consensus recommendation from the ECTS rare bone disease action group. Calcif Tissue Int 2025; 116: 73. 40346280 10.1007/s00223-025-01382-w PMC12064599

[15] Gittus M Chong J Sutton A , et al. Barriers and facilitators to the implementation of guidelines in rare diseases: a systematic review. Orphanet J Rare Dis 2023; 18: 140. 37286999 10.1186/s13023-023-02667-9 PMC10246545

[16] Cunningham CT Quan H Hemmelgarn B , et al. Exploring physician specialist response rates to web-based surveys. BMC Med Res Methodol 2015; 15: 32. 25888346 10.1186/s12874-015-0016-z PMC4404667

[17] Wren TA Gorton GE 3rd Ounpuu S , et al. Efficacy of clinical gait analysis: a systematic review. Gait Posture 2011; 34: 149–153. 21646022 10.1016/j.gaitpost.2011.03.027

[18] Monig I Steenvoorden D de Graaf JP , et al. CPMS-improving patient care in Europe via virtual case discussions. Endocrine 2021; 71: 549–554. 33528763 10.1007/s12020-021-02628-x PMC7851636

[19] Fortunato F Bianchi F Ricci G , et al. Digital health and Clinical Patient Management System (CPMS) platform utility for data sharing of neuromuscular patients: the Italian EURO-NMD experience. Orphanet J Rare Dis 2023; 18: 196. 37480080 10.1186/s13023-023-02776-5 PMC10360326

[20] Crowley R Wolfe I Lock K , et al. Improving the transition between paediatric and adult healthcare: a systematic review. Arch Dis Child 2011; 96: 548–553. 21388969 10.1136/adc.2010.202473

[21] Celli L Garrelfs MR Sakkers RJB , et al. Adapting to adulthood: a review of transition strategies for osteogenesis imperfecta. Calcif Tissue Int 2024; 115: 960–975. 39535563 10.1007/s00223-024-01305-1 PMC11607004

[22] Babac A von Friedrichs V Litzkendorf S , et al. Integrating patient perspectives in medical decision-making: a qualitative interview study examining potentials within the rare disease information exchange process in practice. BMC Med Inform Decis Mak 2019; 19: 188. 31533712 10.1186/s12911-019-0911-z PMC6751820

[23] Szlamka Z Tekola B Hoekstra R , et al. The role of advocacy and empowerment in shaping service development for families raising children with developmental disabilities. Health Expect 2022; 25: 1882–1891. 35644908 10.1111/hex.13539 PMC9327816

[24] Boarini M Tremosini M Di Cecco A , et al. Health-related quality of life and associated risk factors in patients with Multiple Osteochondromas: a cross-sectional study. Qual Life Res 2024; 33: 1323–1334. 38457053 10.1007/s11136-024-03604-4 PMC11045590

[25] Guillen-Navarro E AlSayed M Alves I , et al. Recommendations for management of infants and young children with achondroplasia: does clinical practice align? Orphanet J Rare Dis 2025; 20: 114. 40065464 10.1186/s13023-025-03621-7 PMC11895228

[bibr26-18632521261439074] Boero S Vodopiutz J Maghnie M , et al. International expert opinion on the considerations for combining vosoritide and limb surgery: a modified delphi study. Orphanet J Rare Dis 2024; 19: 347. 39289684 10.1186/s13023-024-03236-4 PMC11409630

[27] Westerheim I Cormier-Daire V Gilbert S , et al. Osteogenesis imperfecta: a study of the patient journey in 13 European countries. Orphanet J Rare Dis 2024; 19: 331. 39252130 10.1186/s13023-024-03345-0 PMC11386111

[28] Trisolino G Depaoli A Menozzi GC , et al. Virtual surgical planning and patient-specific instruments for correcting lower limb deformities in pediatric patients: preliminary results from the in-office 3d printing point of care. J Pers Med 2023; 13: 20231128. 10.3390/jpm13121664 PMC10745053 38138890

[29] Benady A Gortzak Y Ovadia D , et al. Advancements and applications of 3D printing in pediatric orthopedics: a comprehensive review. J Child Orthop 2025; 19: 119–138. 40098806 10.1177/18632521251318552 PMC11910743

