# Supplementary material for: Clinical decision-making in rare bone diseases – A survey among members of the European Paediatric Orthopaedic Society (EPOS) and the European Reference Network on Rare Bone Diseases (ERN BOND)
Source: J Child Orthop. 2026 Apr 21;20(4):428–39. doi: 10.1177/18632521261439074 (PMC13099750; doi:10.1177/18632521261439074)
Supplement: sj-pdf-2-cho-10.1177_18632521261439074 – Supplemental material for Clinical decision-making in rare bone diseases – A survey among members of the European Paediatric Orthopaedic Society (EPOS) and the European Reference Network on Rare Bone Diseases (ERN BOND) [file sj-pdf-2-cho-10.1177_18632521261439074.pdf]

Survey on Clinical Decision Making in Rare Bone Diseases

Dear all

We would like to invite you to participate in a survey on "Clinical decision-making in rare bone diseases".

The survey is designed to target healthcare professionals involved in clinical care, research, decision-making or assessment, or have other experience working with rare bone diseases. This survey will gather insights into the current clinical practices, decision-making strategies, interdisciplinary collaboration, transition in care and future perspectives in the management of rare bone diseases. This initiative supports the mission of the European Reference Network: *"To define a common minimum standard for the assessment of patients with rare bone diseases across European borders.*

This survey gives the opprtunity to share your experiences, challenges and your future perspectives in the assessment of patients with rare bone diseases. By addressing a broad range of relevant topics, we hope to encourage wide participation as it is essential to ensure we gather diverse, relevant, and representative information.Your insights will play a vital role in shaping future standards of care for these complex and often underrepresented conditions.

The results will assist in the development of recommendations, guidelines, and educational tools, aimed at healthcare providers across Europe to support better outcomes for patients and their families.

Your participation in this survey is voluntary. Your responses will remain anonymous and no personally identifiable information will be collected. You can choose to end the survey at any point. Data will be saved only when you submit the responses at the end of the survey. Therefore, if you wish to submit your response, please make sure to do so at the end of the survey.

Please answer each question based on your experience and knowledge.

Completion of the survey implies informed consent.

Estimated completion time: 10–15 minutes.

* Indicates required question

Screening question

1. Do you currently provide, or have you previously provided, clinical care, conducted research, contributed to decision-making or assessment, or have other experience working with rare bone diseases? *

   Mark only one oval.

   - Yes *Skip to question 2*
   - No

Background Information

2. **1. What is your professional background?** *

(Select all that apply)

Check all that apply.

- [ ] Orthopedic surgeon
- [ ] Paediatrician
- [ ] Geneticist
- [ ] Endocrinologist
- [ ] Rheumatologist
- [ ] Radiologist
- [ ] Rehabilitation specialist
- [ ] Physiotherapist
- [ ] Gait analyst
- [ ] Researcher
- [ ] Patient representative
- [ ] Other: ______________________

3. **2. What is your country of practice (Please specify)** *

Dropdown

Mark only one oval.

- Afghanistan
- Albania
- Algeria
- Andorra
- Angola
- Antigua and Barbuda
- Argentina
- Armenia
- Australia
- Austria
- Azerbaijan
- Bahamas
- Bahrain
- Bangladesh
- Barbados
- Belarus
- Belgium
- Belize
- Benin
- Bhutan
- Bolivia
- Bosnia and Herzegovina
- Botswana
- Brazil
- Brunei
- Bulgaria
- Burkina Faso
- Burundi
- Cabo Verde
- Cambodia
- Cameroon
- Canada
- Central African Republic
- Chad
- Chile
- China
- Colombia
- Comoros
- Congo (Congo-Brazzaville)
- Costa Rica
- Croatia

- Cuba
- Cyprus
- Czech Republic
- Democratic Republic of the Congo
- Denmark
- Djibouti
- Dominica
- Dominican Republic
- Ecuador
- Egypt
- El Salvador
- Equatorial Guinea
- Eritrea
- Estonia
- Eswatini
- Ethiopia
- Fiji
- Finland
- France
- Gabon
- Gambia
- Georgia
- Germany
- Ghana
- Greece
- Grenada
- Guatemala
- Guinea
- Guinea-Bissau
- Guyana
- Haiti
- Honduras
- Hungary
- Iceland
- India
- Indonesia
- Iran
- Iraq
- Ireland
- Israel
- Italy
- Jamaica
- Japan
- Jordan

- Kazakhstan
- Kenya
- Kiribati
- North Korea
- South Korea
- Kuwait
- Kyrgyzstan
- Laos
- Latvia
- Lebanon
- Lesotho
- Liberia
- Libya
- Liechtenstein
- Lithuania
- Luxembourg
- Madagascar
- Malawi
- Malaysia
- Maldives
- Mali
- Malta
- Marshall Islands
- Mauritania
- Mauritius
- Mexico
- Micronesia
- Moldova
- Monaco
- Mongolia
- Montenegro
- Morocco
- Mozambique
- Myanmar
- Namibia
- Nauru
- Nepal
- The Netherlands
- New Zealand
- Nicaragua
- Niger
- Nigeria
- North Macedonia
- Norway

- Oman
- Pakistan
- Palau
- Palestine
- Panama
- Papua New Guinea
- Paraguay
- Peru
- Philippines
- Poland
- Portugal
- Qatar
- Romania
- Russia
- Rwanda
- Saint Kitts and Nevis
- Saint Lucia
- Saint Vincent and the Grenadines
- Samoa
- San Marino
- Sao Tome and Principe
- Saudi Arabia
- Senegal
- Serbia
- Seychelles
- Sierra Leone
- Singapore
- Slovakia
- Slovenia
- Solomon Islands
- Somalia
- South Africa
- South Sudan
- Spain
- Sri Lanka
- Sudan
- Suriname
- Sweden
- Switzerland
- Syria
- Taiwan
- Tajikistan
- Tanzania
- Thailand

- Timor-Leste
- Togo
- Tonga
- Trinidad and Tobago
- Tunisia
- Turkey
- Turkmenistan
- Tuvalu
- Uganda
- Ukraine
- United Arab Emirates
- United Kingdom
- United States
- Uruguay
- Uzbekistan
- Vanuatu
- Vatican City
- Venezuela
- Vietnam
- Yemen
- Zambia
- Zimbabwe
- Other

4. **3. What is your primary place of practice?** *

(Select one)

Mark only one oval.

- University hospital/ Academic centre
- General hospital
- Specialty hospital
- Private practice
- Research institution
- Rehabilitation centre
- Other: ____________________

5. **4. How are you involved in the care or management of patients with rare bone diseases?** *

(Select all that apply)

Check all that apply.

- [ ] I provide direct clinical care (diagnosis, treatment, or management)
- [ ] I conduct clinical research on rare bone diseases
- [ ] I am involved in laboratory/genetic testing related to these conditions
- [ ] I am involved in rehabilitation or physiotherapy for affected patients
- [ ] I am involved in imaging/biomechanical evaluation (e.g., gait analysis)
- [ ] I support patients through advocacy, education, or counselling
- [ ] I work in a policy, regulatory, or funding role related to rare diseases
- [ ] I do not currently work with patients with rare bone diseases
- [ ] Other: ____________________

6. **5. Experience and Expertise** *

5. 1 How many years of experience do you have in working with patients with rare bone diseases?

Mark only one oval.

- ( ) less than 2 years
- ( ) 2 - 5 years
- ( ) 6 -10 years
- ( ) 11-15 years
- ( ) more than 15 years
- ( ) You are a patient representative

7. **5. 2 Please indicate the type(s) of rare bone diseases you have experience working with:** *

(Select all that apply)

Check all that apply.

- [ ] Osteogenesis imperfecta
- [ ] Achondroplasia
- [ ] Fibrous dysplasia
- [ ] X-linked hypophosphatemia
- [ ] Fibrodysplasia ossificans progressiva
- [ ] Hypophosphatasia
- [ ] Multiple osteochondromas
- [ ] None of the above
- [ ] Other: ____________________

Diagnostic Work-Up and Decision-Making

(Select one option per item: Routinely, As needed, Never, Not Applicable)

6. What clinical examinations do you routinely use in patients with rare bone diseases?
(Select all that apply)

8. **6.1 Growth parameters** *

Mark only one oval per row.

	Not Applicable	Never	As needed	Routinely
**Height**	⬭	⬭	⬭	⬭
**Sitting height**	⬭	⬭	⬭	⬭
**Weight**	⬭	⬭	⬭	⬭
**Growth chart**	⬭	⬭	⬭	⬭
**Body proportions (arm span vs height)**	⬭	⬭	⬭	⬭
**BMI**	⬭	⬭	⬭	⬭
**Other (please specify below)**	⬭	⬭	⬭	⬭

9. 6.1.1 Please specify what other "**growth parameters**" you use when examining patients with rare bone diseases:

____

10. **6.2 Posture and alignment in standing position** *

Mark only one oval per row.

	Not Applicable	Never	As needed	Routinely
**Pelvic tilt**	⬭	⬭	⬭	⬭
**Shoulder height**	⬭	⬭	⬭	⬭
**Head position**	⬭	⬭	⬭	⬭
**Inspection of the spine (scoliosis, kyphosis, lordosis)**	⬭	⬭	⬭	⬭
**Forward bend test to look for paraspinal prominence**	⬭	⬭	⬭	⬭
**Other (please specify below)**	⬭	⬭	⬭	⬭

11. 6.2.1 Please specify what other "**measurements of posture and alignment in standing position**" you use when examining patients with rare bone diseases:

____

12. **6.3 Posture and alignment in sitting position** *

Mark only one oval.

⬭ Yes

⬭ No

⬭ Not applicable

13. **6.4 Observation of gait** *

Mark only one oval per row.

	Not Applicable	Never	As needed	Routinely
**Symmetry**	⬭	⬭	⬭	⬭
**Rhythm and cadence**	⬭	⬭	⬭	⬭
**Speed and stride length**	⬭	⬭	⬭	⬭
**Rotational profile**	⬭	⬭	⬭	⬭
**Lower limb deformities, contractures, instabilities**	⬭	⬭	⬭	⬭
**Trendelenburg**	⬭	⬭	⬭	⬭
**Trunk lean**	⬭	⬭	⬭	⬭
**Other (please specify below)**	⬭	⬭	⬭	⬭

14. 6.4.1 Please specify <u>what other</u> "**observational gait analyses**" you use when examining patients with rare bone diseases:

____

15. **6.5 Range of motion (ROM) testing** *

Mark only one oval per row.

	Not Applicable	Never	As needed	Routinely
**Active and passive ROM in all joints in the lower extremity**				
**Active and passive ROM in all joints in the upper extremity**				
**Testing of joint instability**				
**Other (please specify below)**				

16. 6.5.1 Please specify what other "**Range of motion (ROM) parameters**" you use when examining patients with rare bone diseases:

17. **6.6 Other tests, scores, questionnaires** *

Mark only one oval per row.

	Not Applicable	Never	As needed	Routinely
**Muscle strength testing**	⬭	⬭	⬭	⬭
**Muscle mass and tone**	⬭	⬭	⬭	⬭
**Pain assessment**	⬭	⬭	⬭	⬭
**Neurological exam**	⬭	⬭	⬭	⬭
**Any functional mobility tests (e.g. 6-minute walk test, Time up and go) (please specify below)**	⬭	⬭	⬭	⬭
**Any patient reported outcome measures (e.g. VAS, EQ-5D) (please specify below)**	⬭	⬭	⬭	⬭
**Other (please specify below)**	⬭	⬭	⬭	⬭

18. 6.6.1 Please specify which "**functional mobility tests**" you use when examining patients with rare bone diseases: *

____

19. 6.6.2 Please specify which "**patient reported outcome measures (PROMs)**" you use in patients with rare bone diseases: *

____

20. 6.6.3 Please specify if you use any "**other tests, scores or questionnaires**" when examining patients with rare bone diseases:

21. **7. What imaging modalities do you most frequently use in clinical decision making in patients with rare bone diseases?** *

(Select all that apply)

Mark only one oval per row.

	Not Applicable	Never	As needed	Routinely
**Radiographs of bone segment of interest**				
**Long standing radiographs**				
**CT**				
**Weight bearing CT**				
**MRI**				
**DEXA : Spine**				
**DEXA : Hip**				
**DEXA : Total Body**				
**High-Resolution Imaging (HR-pQCT)**				
**EOS**				
**Other (please specify below)**				

22. 7.1 Please specify what other "**imaging modalities**" you use:

23. **8. Which biochemical/laboratory tests do you routinely use in clinical decision making in patients with rare bone diseases?** *

(Select all that apply)

Mark only one oval per row.

	Not Applicable	Never	As needed	Routinely
**Serum calcium**	⬭	⬭	⬭	⬭
**Phosphate**	⬭	⬭	⬭	⬭
**ALP (Alkaline Phosphatase)**	⬭	⬭	⬭	⬭
**PTH (Parathyroid Hormone)**	⬭	⬭	⬭	⬭
**Vitamin D (25-OH)**	⬭	⬭	⬭	⬭
**Genetic testing (targeted or whole exome/genome)**	⬭	⬭	⬭	⬭
**Bone turnover markers**	⬭	⬭	⬭	⬭
**Other (please specify below)**	⬭	⬭	⬭	⬭

24. 8.1 Please specify <u>what other</u> "**biochemical/laboratory tests**" you use in addition to the above metioned:

____

25. **8.2 Which of the following bone turnover markers do you test in patients with rare bone diseases?** *

(Select all that apply)

Check all that apply.

- [ ] Procollagen Type I N-Propeptide (P1NP)
- [ ] C-terminal telopeptide of type I collagen (CTX)
- [ ] Bone-specific alkaline phosphatase (BALP)
- [ ] Other: ____

26. **9. Do you have an interdisciplinary approach in clinical decision making in patients with rare bone diseases?** *

Mark only one oval.

- Always
- As needed
- Never

27. **10. Which specialities are most often involved in interdisciplinary care for rare bone diseases at your institution?** *

(Select all that apply)

Check all that apply.

- Orthopaedics
- Endocrinology
- Paediatrician
- Genetics
- Radiology
- Rehabilitation specialist
- Neurology
- Rheumatology
- Psychlogist
- Physiotherapist
- Occupational therapist
- Interdisciplinary care not offered
- Other: ____________________

28. **11. How frequently are interdisciplinary case discussions of complex rare bone diseases cases held?** *

Mark only one oval.

- Weekly
- Monthly
- Occasionally
- Never
- Other: ____________________

Research, Natural History and Long-term data

29. **12. Do you have access to natural history data or long-term follow-up studies or registries for patients with rare bone diseases?** *

Mark only one oval.

- Yes, national registry
- Yes, international registry
- Local/institutional database
- Long-term follow up studies from available literature
- No access

30. **13. How important do you find long-term follow-up studies and natural history studies for guiding treatment decisions in childhood and adolescence?** *

Mark only one oval.

	1	2	3	4	5	
Not	◯	◯	◯	◯	◯	Extremely Important

31. **14. Are long-term outcomes (e.g. growth, function, quality of life) considered in your clinical decision-making?** *

Mark only one oval.

	1	2	3	4	5	
Neve	◯	◯	◯	◯	◯	Always

Role of Gait Analysis

32. **15. Which types of gait analysis do you use in patients with rare bone diseases?** *

(Select all that apply)

Check all that apply.

- [ ] Fully instrumented gait analysis (FGA)
- [ ] 3D motion capture
- [ ] Wearable sensors
- [ ] Video-based analysis (AI/deep learning)
- [ ] None

33. **16. How often do gait analysis findings directly influence your treatment decisions in patients with rare bone diseases?** *

Mark only one oval.

	1	2	3	4	5	
Neve	◯	◯	◯	◯	◯	Always

34. **17. Evaluate the level of effectiveness of gait analysis to assess the clinical outcomes listed below in patients with rare bone diseases:** *

Mark only one oval per row.

	Not sure/Not applicable	Ineffective	Neutral	Effective	Very effective
**Surgical planning**	⬭	⬭	⬭	⬭	⬭
**Monitoring rehabilitation**	⬭	⬭	⬭	⬭	⬭
**Identifying compensatory movements**	⬭	⬭	⬭	⬭	⬭
**Understanding functional limitations**	⬭	⬭	⬭	⬭	⬭
**Research**	⬭	⬭	⬭	⬭	⬭

35. **18. What is the main clinical benefit of gait analysis in rare bone diseases?** *

(Select all that apply)

Check all that apply.

- [ ] Surgical planning
- [ ] Monitoring rehabilitation
- [ ] Identifying compensatory movements
- [ ] Understanding functional limitations
- [ ] Research only
- [ ] Other: ____________________

36. **19. What are the main limitations of using gait analysis in clinical settings?** *

(Select all that apply)

Check all that apply.

- [ ] Cost and access
- [ ] Time/resource limitations
- [ ] Lack of guidelines and standardised protocols
- [ ] Limited relevance for some rare bone disease types
- [ ] Patient compliance
- [ ] Other: ____________________

37. **20. Which patient subgroups do you think benefit the most from gait analysis in the context of rare bone diseases?** *

(Select all that apply)

Check all that apply.

- [ ] Patients with lower limb deformities
- [ ] Patients with moderate to severe impact in mobility and with postural control issues
- [ ] Patients undergoing surgical treatment (for pre- and post-assessment)
- [ ] Patients with discrepancies between static imaging and dynamic gait analysis
- [ ] Patients with rapidly progressing RBDs
- [ ] Patients with significant functional impairment due to RBD
- [ ] All RBD patients
- [ ] Other: ____________________

Future Perspectives

38. **21. To what extent do you agree with the following:** *

"Better integration of clinical, imaging, and functional data (e.g., gait analysis) would improve clinical decision-making in RBDs."

Mark only one oval.

	1	2	3	4	5	
Stro	◯	◯	◯	◯	◯	Strongly agree

39. **22. To what extend do you use digital planning and 3D printing to better understand deformities and to guide treatment?** *

Mark only one oval per row.

	Never	Rarely	Sometimes	Often	Always
**Digital planning**	◯	◯	◯	◯	◯
**3D printing**	◯	◯	◯	◯	◯

40. **23. Which innovations do you think will shape the future of rare bone disease management?** *

(Select up to 3)

Check all that apply.

- [ ] AI-based imaging interpretation
- [ ] Digital twins/ virtual modelling
- [ ] Remote monitoring tools
- [ ] Standardized care pathways
- [ ] Cross-disciplinary guidelines
- [ ] Patient-reported outcome tools
- [ ] Other: ____________________

Shared/Patient-centred decision making

41. **24. In your opinion, how valuable is the role of patient representatives in clinical decision-making?** *

Mark only one oval.

- Very valuable
- Somewhat valuable
- Neutral
- Limited
- Not involved

42. **25. To what extent do you consider the following socio-cultural values and preferences of patients with rare bone diseases during clinical decision making?** *

Mark only one oval per row.

	Not at all	Slightly	Moderately	Very much	Completely
**Overall quality of life**					
**Patient goals and expectations**					
**Tolerance or concerns about specific treatments**					
**Cultural or religious beliefs**					
**Socioeconomic status**					
**Family or caregiver input**					
**Long-term impact of treatment decisions**					
**Patient-reported outcomes and feedback**					
**Emotional or mental health considerations**					
**Other (please specify below)**					

43. Please specify what other "**cultural and social values and preferences of patients**" are considered in the clinical decision making of patients with rare bone disseases:

____

44. **26. How would you rate your experience collaborating with patients and families in managing their rare bone disease?** *

Mark only one oval.

	1	2	3	4	5	
Very	◯	◯	◯	◯	◯	Very effective

45. **27. What tools or resources would enhance your ability to engage patients and families in shared decision-making for rare bone disease?** *

(Select all that apply)

Check all that apply.

- ☐ Time and resource support to facilitate shared decision-making
- ☐ Access to multidisciplinary input (e.g., geneticists, psychologists, social workers)
- ☐ Evidence-based decision aids (e.g., visual tools comparing treatment options)
- ☐ Patient education materials (e.g., brochures, videos, translated resources)
- ☐ Translated educational materials or culturally adapted resources
- ☐ Training in shared decision making communication methods (e.g., Three-Talk Model, BRAN, SHARE)
- ☐ Involve patient organizations and participate in patient advocacy meetings
- ☐ Other: ____

Transition of Care from pediatric to adult services

46. **28. Do you or your institution provide continuity of care (transition care) from paediatric to adult services for patients with rare bone diseases?** *

Mark only one oval.

- ◯ Yes, we have a formal transition care program
- ◯ Yes, we provide continuous care without a formal transition care program
- ◯ No, patients are referred to other services

47. **29. How important do you consider the need for a formal structured tool for continuity of care (transition care) in rare bone disease patients?** *

Mark only one oval.

	1	2	3	4	5	
Not	◯	◯	◯	◯	◯	Extremely important

48. **30. What challenges do you often face when transitioning patients with rare bone diseases from paediatric to adult care?** *

(Select all that apply)

Check all that apply.

- [ ] Poor communication/collaboration between paediatric and adult teams
- [ ] Limited transition guidelines or protocols
- [ ] Loss of follow-up or patient disengagement
- [ ] Geographic barriers (e.g., different geographic locations, long travel distances to adult centres)
- [ ] Limited availability of multidisciplinary teams in adult services
- [ ] Other: ____________________

Closing remarks and suggestions

49. **31. Do you have any additional comments, suggestions, or insights regarding clinical decision making in rare bone diseases or the topics addressed in this survey?** *

____

____

____

____

____

Closing Statement

Thank you for your valuable input!

Your experience and perspectives are crucial for guiding future improvements in care, collaboration, and clinical decision-making in rare bone diseases.

This content is neither created nor endorsed by Google.

Google Forms
